# The role of supervisor support in the association between night work and depressive symptoms: a gender-stratified analysis of 22,422 full-time wage workers in Korea

**DOI:** 10.4178/epih.e2024079

**Published:** 2024-09-25

**Authors:** Hee Won Kim, Ji-Hwan Kim, Garin Lee, Hye-Lin Lee, Hayoung Lee, Seung-Sup Kim

**Affiliations:** 1 Department of Environmental Health Sciences, Graduate School of Public Health, Seoul National University, Seoul, Korea; 2 Institute of Health and Environment, Seoul National University, Seoul, Korea

**Keywords:** Depression, Mental health, Shift work schedule, Psychosocial support systems, Social support

## Abstract

**OBJECTIVES:**

This study investigated the relationship between night work, supervisor support, and depressive symptoms among full-time wage workers, with a focus on gender differences.

**METHODS:**

A nationwide sample of 22,422 full-time wage workers from the Sixth Korean Working Conditions Survey (2020-2021) was analyzed. Experiences of night work were categorized into 5 groups based on the number of night work days per month: 0, 1-5, 6-10, 11-15, and 16-31. Depressive symptoms were evaluated using the 5-item World Health Organization Well-Being Index. Supervisor support was assessed with 5 items.

**RESULTS:**

Workers who engaged in 1-5 days (prevalence ratio [PR], 1.23; 95% confidence interval [CI], 1.12 to 1.36) and 6- 10 days (PR, 1.17; 95% CI, 1.06 to 1.30) of night work per month exhibited a higher prevalence of depressive symptoms than those without night work. After stratifying by supervisor support levels, workers with 1-5 days, 6-10 days, and 11-15 days of night work per month were more likely to experience depressive symptoms compared to those without night work in the low supervisor support group. In contrast, no association was found between night work (≥6 days) and depressive symptoms in the high supervisor support group. Furthermore, gender differences were notable: female workers with 6-10 days (PR, 1.45; 95% CI, 1.23 to 1.70), and 11-15 days (PR, 1.43; 95% CI, 1.08 to 1.90) of night work per month exhibited a higher prevalence of depressive symptoms, whereas their male counterparts did not. This pattern of gender difference was also found among those with low supervisor support.

**CONCLUSIONS:**

Supervisor support may mitigate the adverse effects of night work on depressive symptoms among full-time wage workers, with differences manifested across genders.

## GRAPHICAL ABSTRACT

[Fig f1-epih-46-e2024079]

## Key Message

Night workers play a crucial role in society yet face unique mental health challenges. While a growing body of research have focused on their health problems, the role of supervisor support remains unexplored. Using a nationally representative dataset of workers in Korea, we found that night work was associated with depressive symptoms, especially for those working 1-10 nights monthly and for female workers conducting 6-15 days of night work. Strong supervisor support may modify these effects. Our findings underscore the need for organizations to acknowledge the adverse mental health consequences of night work and to foster a supportive workplace culture.

## INTRODUCTION

Night work, as defined by the World Health Organization (WHO), is any work performed during the regular sleeping hours of the general population [1]. These hours, typically recognized as being from 23:00 to 07:00, may vary slightly among different communities [1]. Night work forms an essential part of shift work, which involves employees taking turns to ensure that the organization continues to operate outside the usual working hours of any single worker [2]. Globally, night workers constitute a significant segment of the workforce, accounting for 14% of workers in European Union (EU) countries, 7.4% in the United States, and 9.8% in Korea [3-5].

A growing body of research suggests that night work is a risk factor for various mental disorders, including anxiety, mood disorders, and depression [6-8]. Previous studies have primarily explored the relationship between night work and depressive symptoms. For example, a meta-analysis of 11 observational studies found a statistically significant association between night work and depression [9]. Furthermore, a prospective study involving 175,543 workers in the United Kingdom found that, compared to workers who had never worked shifts, those who engaged in occasional or fixed shifts (including night work) had hazard ratios for depression that were 1.23 times and 1.21 times higher, respectively [10].

In Korea, females predominantly handle domestic tasks, a practice deeply rooted in the enduring influence of the Confucian patriarchal system [11]. A nationwide study in Korea found that in households where females are the sole income earners, they spend an average of 156 minutes per day on housework, while males spend about 119 minutes. In households where both partners earn, females spend 187 minutes daily on domestic duties, whereas males contribute just 54 minutes [12]. This pattern holds true regardless of females’ employment status, with females consistently performing the majority of household tasks. Social perceptions continue to reinforce this imbalance in gender roles, with only a small minority across genders believing that males should equally share or assume responsibility for household chores [13]. This disparity leads to work-family conflict, especially for females with irregular and non-standard work hours.

Social support is known to promote positive workplace experiences. Social support is typically defined as a coping resource that individuals use to manage various types of stressors [14]. Unlike the quantitative assessment of social relationships, such as social network size, social support is a qualitative measure of social interactions. In the workplace, this support can come from colleagues, supervisors, and the organization itself [15]. Notably, high levels of support from supervisors can act as a protective or buffering factor against mental health issues such as stress, anxiety, and depression [16]. For instance, a study involving 8,833 workers from the Maastricht cohort found that male employees who reported lower levels of supervisor support had 1.38 times higher odds of experiencing fatigue compared to those with high supervisor support [17]. Similarly, the Whitehall II study revealed that among male white-collar workers, those with low levels of supervisor support were at a higher risk of developing psychiatric disorders in the follow-up analysis [18].

Prior research examining the correlation between experiences of shift (including night) work and depressive symptoms has largely treated supervisor support as a confounder rather than an effect modifier. Findings from the Maastricht cohort study indicate that male shift workers aged 45 and older faced higher risks of developing depressive symptoms after adjusting for multiple confounders, including supervisor support [19]. Similarly, another cohort study controlled for leader and colleague support when exploring the relationship between shift work and subjective well-being [20]. In contrast, studies have highlighted the protective role of social support on mental health outcomes among workers with various work-related experiences, suggesting that social support should not be treated merely as a confounding factor to be controlled for [21].

While the mental health effects of night work have been extensively studied, previous research has not examined the role of supervisor support in the relationship between night work and depressive symptoms among Korean full-time wage workers, nor has it investigated such variations based on gender. To address these gaps in knowledge, this study aimed to explore the following questions: (1) Is night work associated with a higher prevalence of depressive symptoms? (2) Does the level of supervisor support modify the relationship between night work and depressive symptoms? (3) Does this association vary by gender?

## MATERIALS AND METHODS

### Study population

Our study utilized data from the Sixth Korean Working Conditions Survey (KWCS) conducted during 2020-2021 [22]. This survey, which benchmarked the European Working Conditions Survey (EWCS), aimed to explore occupational risk factors in the workplace and assess the overall health of workers. The original dataset comprised of 50,538 actively employed individuals. However, our analysis was restricted to 25,735 full-time wage workers after excluding part-time workers (n=7,165), self-employed individuals (n=15,870), unpaid family workers (n=1,605), and cases with missing data (n=163). The final analysis included 22,422 full-time wage workers, following the exclusion of missing data related to independent (n=52), dependent (n=44), and effect modifier variables (n=1,578), as well as potential confounders (n=1,639).

### Measures

#### Number of days of night work per month

Experiences of night work were assessed with the question: “Normally, how many times a month do you work at night (for at least 2 hours between 10:00 p.m. and 5:00 a.m.)?” Participants could respond on a continuous scale. Based on their answers, the number of night work days per month was divided into 5 categories: (1) 0 days; (2) 1-5 days; (3) 6-10 days; (4) 11-15 days; and (5) 16-31 days.

#### Depressive symptoms

Depressive symptoms were analyzed using 5 items from the World Health Organization’s Five Well-Being Index (WHO-5). The questions included: (1) “I have felt cheerful and in good spirits”; (2) “I have felt calm and relaxed”; (3) “I have felt active and vigorous”; (4) “I woke up feeling fresh and rested”; and (5) “My daily life has been filled with things that interest me” (in the last two weeks). Participants rated each item on a 6-point Likert scale, ranging from 1 (“all of the time”) to 6 (“at no time”). The scores were then summed and subtracted from a total of 30 points. According to the WHO-5 guidelines, a raw score of 13 points or below was categorized as indicative of poor subjective mental health or depressive symptoms [23]. Previous studies have established the WHO-5 as a reliable and valid measure for assessing subjective well-being and as a screening tool for depression [24].

#### Supervisor support

Supervisor support levels were assessed using 5 specific items: (1) “Immediate boss respects you as a person”; (2) “Immediate boss is successful in getting people to work together”; (3) “Immediate boss is helpful in getting the job done”; (4) “Immediate boss provides useful feedback on your work”; and (5) “Immediate boss encourages and supports your development.” Participants responded using a 5-point Likert scale, where 1 indicated “strongly agree” and 5 as “strongly disagree.” The scores were initially summed and then subtracted from a total possible score of 25. Supervisor support levels were then dichotomized as high (≥ 15 points) or low (0-14 points), using the mean value as the cut-off point. These questions have been previously employed in studies to measure levels of supervisor support [25].

#### Potential confounders

Demographic factors such as age, gender, and number of household members, along with socioeconomic factors including educational level and monthly income, and workplace factors such as employment status, weekly work hours, type of occupation, and company size were considered as potential confounders in this study. Participants were classified by gender into males and females. Age groups were defined as 15-29 years, 30-39 years, 40-49 years, 50-59 years, and older than 60 years old. The number of household members was divided into 4 categories: 1, 2, 3, and 4 or more members. For socioeconomic variables, educational level was divided into 4 groups: primary or lower level of education, middle school graduate, high school graduate, and college graduate or higher. Monthly income was divided into 4 ranges: less than 2,000,000, 2,000,000-2,999,999, 3,000,000-3,999,999, and 4,000,000 Korean won (KRW) or more. In terms of workplace variables, employment status was classified into 3 types: permanent, temporary, and daily employees. Weekly work hours were grouped into 3 categories: 36-39, 40-52, and over 53 hours. Company size was categorized into 3 groups: 1 to 49, 50 to 299, and 300 or more employees. Finally, occupations were divided into 3 main categories: white-collar workers (administrators, professionals, semi-professionals, office workers), pink-collar workers (service and sales workers), and blue-collar workers (agriculture, forestry, and fishery industry skilled workers, technical skilled workers and related skilled workers, equipment-machinery operators and assembly workers, simple labor workers).

### Statistical analysis

Since the prevalence of depressive symptoms in the total study population exceeded 10%, the odds ratios derived from logistic regression analysis might overestimate the results [26]. Consequently, we utilized Poisson regression models with robust error variance to explore the relationship between night work and depressive symptoms, taking into account potential confounders and examining any gender differences.

To determine whether the relationship between depressive symptoms and night work varied with levels of supervisor support, we tested the interaction between each level of supervisor support and the number of night work days per month for statistical significance. We also employed the likelihood ratio test to compare the main effects model, which excludes the interaction term, with the full model that includes it, in order to identify any significant differences. Subsequently, we investigated the relationship between night work and depressive symptoms, after stratifying the data by levels of supervisor support. Additionally, we performed further analysis to explore potential gender differences in this association. Prevalence ratios (PRs) were presented along with their 95% confidence intervals (CIs). Confounders were adjusted for as categorical variables in the models. All statistical analyses were performed using Stata/SE version 17.0 (StataCorp, College Station, TX, USA).

### Ethics statement

This study utilized publicly available data with permission from the Korea Occupational Safety and Health Agency (KOSHA). Informed consent was not necessary for the use of this dataset, as it involved a secondary analysis of publicly available data. The study was exempt from Institutional Review Board approval by Seoul National University (IRB No. E23210/003-003).

## RESULTS

Table 1 presents the distribution of the study population and the prevalence of night work and depressive symptoms by confounders. Among the final study population of 22,422 full-time wage workers in Korea, 9.3% reported engaging in night work for at least 1 day per month. Experiences of night work (≥ 1days) were more common among males, individuals over 60 years old, those with 2 household members, middle school graduates, temporary employees, those working more than 53 hr/wk, employees in companies with over 300 workers, and blue-collar workers (Table 1).

Furthermore, 29.5% of employees reported depressive symptoms. Depressive symptoms were more commonly reported among individuals older than 60 years, those with a primary or lower level of education, those earning a monthly income of less than 2,000,000 KRW, daily laborers, employees working more than 53 hr/wk, those employed in companies with fewer than 49 employees, and blue-collar workers (Table 1).

Table 2 illustrates the association between various frequencies of night work and depressive symptoms. In the adjusted model, workers who engaged in night work for 1-5 days and 6-10 days per month had a 1.23-times and 1.17-times higher prevalence of depressive symptoms, respectively, than those who did not work at night. However, the associations between 11-15 days and 16-31 days of night work per month and depressive symptoms were not statistically significant after adjusting for confounders.

Table 3 illustrates the relationship between the frequency of night work and the prevalence of depressive symptoms, with data stratified by gender. Among males, only those conducting 1-5 days of night work per month exhibited a 1.22-fold increase in the prevalence of depressive symptoms. In contrast, females conducting 1-5 days, 6-10 days, and 11-15 days of night work per month all had a statistically significant association with depressive symptoms. Notably, females undertaking 6-10 and 11-15 night work per month experienced a 1.45-fold and 1.43-fold higher prevalence of depressive symptoms, respectively, compared to those not engaged in any night work.

Likelihood ratio tests indicated a significant difference between the main effects model (without the interaction term) and the full model (which includes the supervisor support× night work interaction terms), after adjusting for covariates (Supplementary Material 1). A statistically significant outcome from the likelihood ratio test implies that the full model, incorporating the interaction term, provides a better fit than the reduced model. Additionally, before stratifying by levels of supervisor support in the Poisson regression model, interaction terms were assessed for statistical significance (Supplementary Material 1). Table 4 presents the results concerning the relationship between night work and depressive symptoms, stratified by levels of supervisor support.

In the group with high supervisor support, individuals who engaged in 1-5 days of night work per month exhibited a higher prevalence of depressive symptoms (PR, 1.20; 95% CI, 1.02 to 1.43) than non-night workers. However, no statistically significant associations were found between workers performing more than 6 days of night work per month and depressive symptoms in the high supervisor support group.

Conversely, among the group experiencing low levels of supervisor support, workers who engaged in 1-5 days (PR, 1.20; 95% CI, 1.07 to 1.35), 6-10 days (PR, 1.20; 95% CI, 1.06 to 1.35), and 11-15 days (PR, 1.18; 95% CI, 1.02 to 1.37) of night work per month were more likely to report depressive symptoms than those with no night work. Interestingly, no statistically significant correlation was found between workers who engaged in more than 16 days of night work per month and depressive symptoms (Table 5).

Furthermore, we assessed the presence of gender differences in the relationship between night work and depressive symptoms after stratifying by supervisor support levels. Among female workers with high supervisor support, those who completed 6-10 days of night work per month exhibited a 1.39 times higher prevalence of depressive symptoms than those who did not conduct night work. On the other hand, no association was found between night work and depressive symptoms among male workers with high supervisor support. Among male workers with low supervisor support, those engaged in 1-5 days of night work per month reported a 1.16 times higher prevalence of depressive symptoms compared to their counterparts who did not conduct night work. Female workers with low supervisor support who undertook 1-5 days (PR, 1.29; 95% CI, 1.06 to 1.56), 6-10 days (PR, 1.43; 95% CI, 1.18 to 1.73), and 11-15 days (PR, 1.40; 95% CI, 1.01 to 1.95) of night work per month all experienced a higher prevalence of depressive symptoms, even after adjusting for covariates.

## DISCUSSION

This study explored the moderating effect of supervisor support on the relationship between night work and depressive symptoms among full-time wage workers in Korea, with a focus on gender differences. Our results show that both male and female workers who conducted 1-5 days of night work per month demonstrated a higher prevalence of depressive symptoms. However, only females with 6-15 days of night work per month were more likely to report depressive symptoms. Furthermore, while the association between engaging in 6-10 days and 11-15 days of night work per month and depressive symptoms was statistically significant among those with low supervisor support, this pattern was not evident among those with high supervisor support. This trend was also observed only among female workers in the low supervisor support group, whereas their male counterparts conducting 6-15 days of night work did not show such pattern.

Previous findings have demonstrated a higher prevalence of depressive symptoms among irregular night workers compared to those with fixed night schedules, which partially aligns with our results. Our analysis, which focused only on full-time wage workers, suggests that employees with fewer night work days per month might indirectly represent the experiences of irregular night workers. A prospective cohort study of British workers found that females with 2 or more years of irregular shift work experience reported higher odds of depressive symptoms compared to those without any night shift work experience [27]. Similarly, another study involving 587 nurses and midwives in the United Kingdom found increased social disruption, sleep disturbances, and job dissatisfaction among nurses on rotating shifts compared to those on permanent shifts [28].

One possible mechanism to explain this phenomenon may be the disruption or misalignment of the internal biological clock among irregular night workers [29]. Since the hormone melatonin is a key regulator of the sleep/wake cycle, a substantial body of literature has focused on the changes in melatonin levels among night workers, particularly highlighting certain differences between fixed and rotating night shifts [30]. For example, Boudreau et al. [31] suggest that circadian entrainment may be possible for individuals exposed to 1 week of fixed night shift work, as evidenced by peak melatonin levels during daytime sleep. This suggests that it may be possible for fixed night shift workers to align their biological clocks with their night work schedules [32]. Another study involving offshore petroleum industry shift workers also reveals that 8 out of 11 workers adjusted their circadian rhythm of melatonin levels within a single week of fixed night shifts [33]. Similar findings were demonstrated in a study of members at the British Antarctic Base of Halley [34]. In fact, the misalignment of the circadian clock has been cited as a prominent feature of major depressive disorder [35]. While it is important to note that these studies suggesting circadian entrainment have been conducted in more isolated research settings and may not be applicable to all types of workplaces, they may serve as a possible mechanism contributing to differences in the prevalence of depressive symptoms among workers with varying frequencies of night work.

In Korea, as of 2019, 29.7% of the 63,863 businesses had adopted a shift work system [36]. Under the Labor Standards Act in Korea, employers are required to pay an additional 50% of the regular wage for work performed between 10:00 p.m. and 6:00 a.m. [37]. This legislation likely prompted the adoption of various forms of stratified shift work systems. A study of 71 Korean manufacturing factories showed that the most common arrangement was rotating 2 groups across 2 shifts (38.0%), followed by rotating 4 groups across 3 shifts (19.7%), and rotating 3 groups across 2 shifts (15.5%) [38]. With many companies implementing these stratified shift work systems, which include irregular night shifts, our findings that indicate an increased risk of depressive symptoms among individuals who engage in 1-10 days of night work per month are particularly relevant. It is noteworthy that both male and female workers who engaged in 1-5 days of night work per month exhibited a higher prevalence of depressive symptoms. This suggests that performing 1-5 days of night work monthly may be particularly detrimental to the mental well-being of workers.

Another important finding of our study is the presence of gender differences in the association between the frequency of night work and depressive symptoms. Research involving full-time wage workers indicates that depressive symptoms are more likely to be reported by females who experience high levels of family-to-work conflict combined with long working hours, whereas those with low levels of conflict do not report these symptoms. In contrast, males with long working hours show a higher prevalence of depressive symptoms, regardless of their level of family-to-work conflict [39]. Work-family conflict, including family-to-work conflict, is a significant workplace stressor, especially for females with non-standard working schedules, such as long working hours and night work. This stressor may explain why females who engage in 6-10 days and 11-15 days of night work per month were more likely to experience depressive symptoms compared to those who do not work at night. A similar pattern was not observed in males. Essentially, females with non-standard working hours, such as night work, may experience higher levels of depressive symptoms, potentially due to the additional stress from work-family conflict.

Another key observation from our study is the potential influence of supervisor support on the relationship between night work experiences and depressive symptoms. We found that depressive symptoms were correlated with experiences of night work in the group with low supervisor support, while these associations were generally not statistically significant in the group with high supervisor support. This observation may be explained by Cohen’s stress-buffering hypothesis, which suggests that social support systems can be beneficial during stressful times by attenuating the stress response system [40].

Previous research has shown that supervisor support plays a crucial role in buffering the mental health of workers. Supervisors can provide instrumental support by offering flexible work schedules or accommodating workers’ needs, which in turn reduces work-family conflict [41]. For example, a study on working parents found that those who had access to instrumental support from their supervisors, including alternative work arrangements, leave time benefits, and stress-management programs, were 2.7 times less likely to experience role strain [42].

In fact, such forms of support provided by supervisors may also help to explain the presence of gender differences observed in the relationship between night work experiences and depressive symptoms by mitigating work-family conflict. A study examining the link between supervisor support and burnout in 343 Spanish workers found that females with adequate supervisor support experienced lower burnout levels, mediated by reduced work-family interference. Conversely, males were less influenced by supervisor support; for them, adequate family support was crucial in reducing burnout levels [43]. These differential impacts of supervisor support on burnout levels among males and females could elucidate our findings, which revealed that among workers with low supervisor support, females who worked 6-10 and 11-15 night work per month reported a higher prevalence of depressive symptoms than those who do not work at night. This association was less evident in males.

Moreover, supervisors are seen as representatives of the company and offer emotional support, playing a crucial role in shaping employees’ perceptions of organizational support. This concept is central to the organizational support theory, which emphasizes the importance of how a company values employee contributions and health [44]. Supervisor support has been shown to be a key predictor of job satisfaction and acts as a mediator for enhanced work engagement through the implementation of work-life balance programs and policies [45-47]. Additionally, a study among females bankers indicated that supervisory emotional support positively correlates with work-life balance [48]. In our study, the 5 items used to assess supervisor support cover both instrumental and emotional support resources, suggesting that these supports could help mitigate the adverse effects of night work on workers’ depressive symptoms.

This study does have certain limitations. First, the cross-sectional design of the KWCS prevents us from determining the temporal relationship between night work and depressive symptoms; thus, we cannot rule out the possibility of reverse causation. Second, the assessment of night work experiences was based on a single question about the frequency of night work per month, which may limit the generalizability of our findings. Although our analysis was confined to full-time wage workers, it is important to recognize that employees with varying frequencies of night work may not align with those having fixed or irregular night work schedules. Additionally, we did not evaluate the total duration of night work experience, preventing us from determining if the observed associations, such as the mental health impacts of night work or the protective role of supervisor support, vary with the length of night work performed. Lastly, it is important to note the lack of a universally accepted definition of night work. Different organizations define night work in various ways: the WHO does not specify a time restriction, the International Labor Organization defines it as work occurring from midnight to 5 a.m. for at least 7 consecutive hours, and the EU categorizes night work as working at least 3 hours of a daily shift from midnight to 5 a.m. [1,49,50]. These discrepancies should be considered when interpreting the findings from our study.

Despite these limitations, our study has clear strengths and valuable implications. First, to the best of our knowledge, this is the first study to explore the impact of supervisor support on the relationship between night work and mental health among workers, while also considering gender differences. Second, we utilized a large, representative sample of Korean workers to analyze our findings. Third, by categorizing night work experiences into different intervals, we have gained a deeper understanding of how varying frequencies of night work may impact the mental health of full-time wage workers, underscoring the importance of such distinctions in future night work research. Lastly, our study underscores the significance of considering supervisor support as an effect modifier in the relationship between night work and depressive symptoms.

In conclusion, this study illustrates that night work is associated with depressive symptoms among full-time wage workers. These results underscore the critical role of robust supervisor support in reducing the mental health risks linked to night work. They also emphasize the necessity for organizations to recognize the adverse mental health impacts of night work and to cultivate a workplace culture that includes targeted interventions based on gender and levels of supervisor support.

## Figures and Tables

**Figure f1-epih-46-e2024079:**
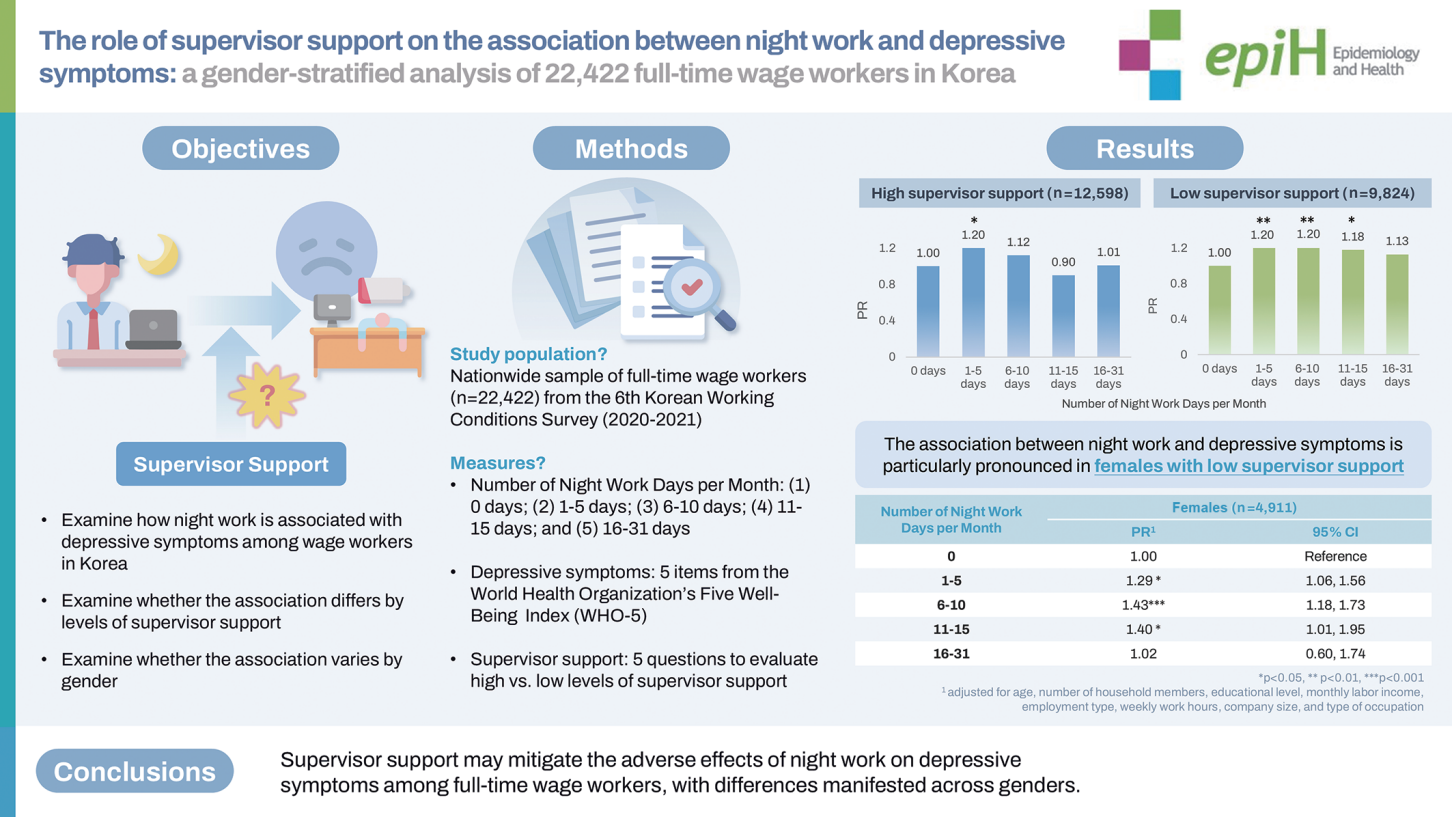


**Table 1.** Distribution of the study population, night work, and depressive symptoms by potential confounders among full-time wage workers in Korea (n=22,422)

Characteristics	Distribution	Night work (≥1 day/mo)	p-value^1^	Depressive symptoms	p-value^2^
Overall	22,422 (100)	2,090 (9.3)		6,611 (29.5)	
Gender			<0.001		0.143
Males	11,691 (52.1)	1,492 (12.8)		3,497 (29.9)	
Females	10,731 (47.9)	598 (5.6)		3,114 (29.0)	
Age (yr)			<0.001		<0.001
15-29	2,932 (13.1)	271 (9.2)		728 (24.8)	
30-39	5,751 (25.7)	490 (8.5)		1,581 (27.5)	
40-49	5,996 (26.7)	484 (8.1)		1,754 (29.3)	
50-59	5,303 (23.7)	476 (9.0)		1,663 (31.4)	
≥60	2,440 (10.9)	369 (15.1)		885 (36.3)	
No. of household members			<0.001		0.225
1	4,615 (20.6)	459 (10.0)		1,383 (30.0)	
2	5,548 (24.7)	571 (10.3)		1,667 (30.1)	
3	5,768 (25.7)	458 (7.9)		1,641 (28.5)	
≥4	6,491 (29.0)	602 (9.3)		1,920 (29.6)	
Educational level			<0.001		<0.001
≤Primary education	309 (1.4)	39 (12.6)		157 (50.8)	
Middle school graduate	869 (3.9)	133 (15.3)		361 (41.5)	
High school graduate	7,407 (33.0)	862 (11.6)		2,464 (33.3)	
≥College graduate	13,837 (61.7)	1,056 (7.6)		3,629 (26.2)	
Monthly income (1,000 Korean won)			0.057		<0.001
<2,000	3,962 (17.7)	380 (9.6)		1,426 (36)	
2,000-2,999	9,057 (40.4)	787 (8.7)		2,722 (30.1)	
3,000-3,999	5,503 (24.5)	534 (9.7)		1,484 (27.0)	
≥4,000	3,900 (17.4)	389 (10.0)		979 (25.1)	
Employment type			0.001		<0.001
Permanent	20,080 (89.6)	1,859 (9.3)		5,738 (28.6)	
Temporary	1,790 (8.0)	198 (11.1)		638 (35.6)	
Daily	552 (2.5)	33 (6.0)		235 (42.6)	
Working hours (hr/wk)			<0.001		<0.001
36-39	368 (1.6)	45 (12.2)		127 (34.5)	
40-52	20,196 (90.1)	1,629 (8.1)		5,767 (28.6)	
≥53	1,858 (8.3)	416 (22.4)		717 (38.6)	
No. of employees			<0.001		0.003
1-49	15,243 (68.0)	1,019 (6.7)		4,599 (30.2)	
50-299	4,704 (21.0)	611 (13.0)		1,336 (28.4)	
≥300	2,475 (11.0)	460 (18.6)		676 (27.3)	
Type of occupation			<0.001		<0.001
White collar	11,463 (51.1)	678 (5.9)		3,071 (26.8)	
Pink collar	4,426 (19.7)	384 (8.7)		1,280 (28.9)	
Blue collar	6,533 (29.1)	1,028 (15.7)		2,260 (34.6)	

Values are presented as number (%).

1 From the chi‐square test comparing the prevalence of night work across different groups.

2 From the chi‐square test comparing the prevalence of depressive symptoms across different groups.

**Table 2.** Association between night work and depressive symptoms among full-time wage workers in Korea (n=22,422)

Night work (day/mo)	Total	Depressive symptoms	Unadjusted model	Adjusted model^1^
0	20,332 (90.7)	5,858 (28.8)	1.00 (reference)	1.00 (reference)
1-5	773 (3.5)	274 (35.5)	1.23 (1.12, 1.36)^***^	1.23 (1.12, 1.36)^***^
6-10	744 (3.3)	262 (35.2)	1.22 (1.11, 1.35)^***^	1.17 (1.06, 1.30)^**^
11-15	356 (1.6)	142 (39.9)	1.38 (1.22, 1.58)^***^	1.09 (0.95, 1.25)
16-31	217 (1.0)	75 (34.6)	1.20 (1.00, 1.44)	1.04 (0.86, 1.25)

Values are presented as number (%) or prevalence ratio (95% confidence interval).

1 Adjusted for gender, age, number of household members, educational level, monthly labor income, employment type, weekly work hours, company size, and type of occupation.

** p<0.01,

*** p<0.001.

**Table 3.** Gender-specific associations between night work and depressive symptoms among Korean full-time wage workers

Night work (day/mo)	Males (n=11,691)			Females (n=10,731)		
	Total (n)	Depressive symptoms, n (%)	PR (95% CI)1	Total (n)	Depressive symptoms, n (%)	PR (95% CI)^1^
0	10,199	2,974 (29.2)	1.00 (reference)	10,133	2,884 (28.5)	1.00 (reference)
1-5	517	186 (36.0)	1.22 (1.08, 1.38)^**^	256	88 (34.4)	1.23 (1.04, 1.45)^*^
6-10	519	167 (32.2)	1.06 (0.93, 1.21)	225	95 (42.2)	1.45 (1.23, 1.70)^***^
11-15	295	115 (39.0)	1.04 (0.88, 1.22)	61	27 (44.3)	1.43 (1.08, 1.90)^*^
16-31	161	55 (34.2)	1.03 (0.83, 1.28)	56	20 (35.7)	1.06 (0.73, 1.53)

PR, prevalence ratio; CI, confidence interval.

1 Adjusted for age, number of household members, educational level, monthly labor income, employment type, weekly work hours, company size, and type of occupation.

* p<0.05,

** p<0.01,

*** p<0.001.

**Table 4.** The role of supervisor support in the association between night work and depressive symptoms among Korean full-time wage workers (n=22,422)

Night work (day/mo)	Total	Depressive symptoms	Unadjusted model	Adjusted model^1^
High supervisor support (n=12,598)				
0	11,472 (91.1)	2,515 (21.9)	1.00 (reference)	1.00 (reference)
1-5	410 (3.3)	107 (26.1)	1.19 (1.01, 1.41)^*^	1.20 (1.02, 1.43)^*^
6-10	416 (3.3)	105 (25.2)	1.15 (0.97, 1.36)	1.12 (0.94, 1.33)
11-15	171 (1.4)	42 (24.6)	1.12 (0.86, 1.46)	0.90 (0.68, 1.19)
16-31	129 (1.0)	34 (26.4)	1.20 (0.90, 1.61)	1.01 (0.76, 1.36)
Low supervisor support (n=9,824)				
0	8,860 (90.2)	3,343 (37.7)	1.00 (reference)	1.00 (reference)
1-5	363 (3.7)	167 (46.0)	1.22 (1.09, 1.37)^**^	1.20 (1.07, 1.35)^**^
6-10	328 (3.3)	157 (47.9)	1.27 (1.13, 1.42)^***^	1.20 (1.06, 1.35)^**^
11-15	185 (1.9)	100 (54.1)	1.43 (1.25, 1.64)^***^	1.18 (1.02, 1.37)^*^
16-31	88 (0.9)	41 (46.6)	1.23 (0.99, 1.55)	1.13 (0.90, 1.42)

Values are presented as number (%) or prevalence ratio (95% confidence interval).

1 Adjusted for gender, age, number of household members, educational level, monthly labor income, employment type, weekly work hours, company size, and type of occupation.

* p<0.05,

** p<0.01,

*** p<0.001.

**Table 5.** Gender-specific association between night work and depressive symptoms among Korean full-time wage workers after stratification by supervisor support

Night work (day/mo)	Males (n=11,691)			Females (n=10,731)		
	Total (n)	Depressive symptoms, n (%)	PR (95% CI)^1^	Total (n)	Depressive symptoms, n (%)	PR (95% CI)^1^
High supervisor support						
0	5,969	1,327 (22.2)	1.00 (reference)	5,503	1,188 (21.6)	1.00 (reference)
1-5	275	75 (27.3)	1.22 (1.00, 1.50)	135	32 (23.7)	1.11 (0.82, 1.50)
6-10	296	66 (22.3)	1.00 (0.80, 1.24)	120	39 (32.5)	1.39 (1.07, 1.81)^*^
11-15	142	32 (22.5)	0.83 (0.60, 1.15)	29	10 (34.5)	1.33 (0.81, 2.17)
16-31	96	23 (24.0)	0.93 (0.65, 1.33)	33	11 (33.3)	1.09 (0.64, 1.84)
Low supervisor support						
0	4,230	1,647 (38.9)	1.00 (reference)	4,630	1,696 (36.6)	1.00 (reference)
1-5	242	111 (45.9)	1.16 (1.01, 1.34)^*^	121	56 (46.3)	1.29 (1.06, 1.56)^*^
6-10	223	101 (45.3)	1.11 (0.95, 1.30)	105	56 (53.3)	1.43 (1.18, 1.73)^***^
11-15	153	83 (54.3)	1.16 (0.98, 1.38)	32	17 (53.1)	1.40 (1.01, 1.95)^*^
16-31	65	32 (49.2)	1.19 (0.93, 1.52)	23	9 (39.1)	1.02 (0.60, 1.74)

PR, prevalence ratio; CI, confidence interval.

1 Adjusted for age, number of household members, educational level, monthly labor income, employment type, weekly work hours, company size, and type of occupation.

* p<0.05,

*** p<0.001.

## Supplementary materials

Supplementary Material 1. Interaction-adjusted prevalence ratios for supervisor support and night work on depressive symptoms
