# Supplementary material for: The role of supervisor support in the association between night work and depressive symptoms: a gender-stratified analysis of 22,422 full-time wage workers in Korea
Source: Epidemiol Health. 2024 Sep 25;46:e2024079. doi: 10.4178/epih.e2024079 (PMC11832242; doi:10.4178/epih.e2024079)
Supplement: Supplementary Material 1. — Interaction-adjusted prevalence ratios for supervisor support and night work on depressive symptoms [file epih-46-e2024079-Supplementary-1.docx]

**Supplementary Materials**

| **Supplementary Material 1. Interaction-adjusted prevalence ratios for supervisor support and night work on depressive symptoms** | | | | |
| --- | --- | --- | --- | --- |
| **Supervisor support level x Night work (days per month)** | **Unadjusted model** | | **Adjusted model ^1^** | |
|  | **PR** | **95% CI** | **PR** | **95% CI** |
| High support x 0 | 1 | Reference | 1 | Reference |
| High Support x 1-5 | 1.19 | 1.01, 1.41 | 1.19* | 1.01, 1.41 |
| High support x 6-10 | 1.15 | 0.97,1.36 | 1.13 | 0.95, 1.34 |
| High support x 11-15 | 1.12 | 0.86,1.46 | 0.94 | 0.72,1.23 |
| High support x 16-31 | 1.20 | 0.90, 1.61 | 1.06 | 0.80, 1.42 |
| Low Support x 0 | 1.72*** | 1,65, 1.80 | 1.68*** | 1.60, 1.75 |
| Low Support x 1-5 | 2.10*** | 1.87, 2.36 | 2.02*** | 1.79, 2.27 |
| Low support x 6-10 | 2.18*** | 1.94, 2.46 | 2.00*** | 1.77, 2.27 |
| Low support x 11-15 | 2.47*** | 2.15, 2.83 | 1.92*** | 1.65, 2.23 |
| Low support x 16-31 | 2.13*** | 1.69, 2.67 | 1.84*** | 1.46, 2.30 |
| PR: prevalence ratio; CI: confidence interval. | | | | |
| *p<0.05, ** p<0.01, ***p<0.001 | | | | |
| ^1^ Adjusted for sex, age, number of household members, educational level, monthly labor income, employment type, weekly workhours, company size and type of occupation | | | | |
| Likelihood-ratio-test between the fully adjusted main effects model (without the interaction terms) and the full model including the supervisor support x night work interaction term: p <0.001 | | | | |
